# Giant intraparietal inguinal hernia misdiagnosed as spigelian hernia in an old woman

**DOI:** 10.11604/pamj.2020.36.117.21652

**Published:** 2020-06-23

**Authors:** Barbara Yordanis Hernandez Cervantes, Radisnay Guzmán Lambert, Duniesky Martínez Lopez, Mariuska Rodríguez Gonzalez, Frank Edwin

**Affiliations:** 1 Department of Surgery, School of Medicine, University of Health and Allied Sciences, Ho, Volta Region, Ghana,; 2 Department of Internal Medicine, School of Medicine, University of Health and Allied Sciences, Ho, Volta Region, Ghana,

**Keywords:** Intraparietal inguinal hernia, Spigelian hernia, giant inguinal hernia

## Abstract

Intraparietal inguinal hernias are a rare variant of inguinal hernia in which the hernia sac lies between the layers of the abdominal muscles. Intraparietal inguinal hernias mimic Spigelian hernias clinically; the diagnosis presents superior difficulties than its treatment. We report a case of a giant intraparietal hernia misdiagnosed as a Spigelian hernia clinically. The patient was 83 years old woman presented with complain of a large swelling over right abdomen for around 25 years. The patient had a huge mass of 25 x 30 cm occupying right flank, right lumbar region extending up to the umbilicus and inguinal region, partially reducible with gurgling sounds. Surgery started with transversal incision over the mass, it was found to be an interstitial variety of intraparietal inguinal hernia with a long viable segment of the small bowel with their mesentery as content of the sac. Hernioplasty with a polypropylene mesh was achieved satisfactorily. The patient was discharged on third postoperative day without complications. It is challenging to diagnose intraparietal hernias preoperatively; intraoperative findings defined its definitive diagnosis and its surgical technique.

## Introduction

The inguinal hernia is one of the most common surgical diseases in clinical practice; giant inguinal hernia, however, is more unusual and significantly challenging in terms of surgical management when it is presented as a rare variant [1]. Intraparietal inguinal hernias are a rare variant of the inguinal hernia in which the hernia sac lies between the layers of the abdominal muscles [1]. The sac of the intraparietal hernia enters the internal inguinal ring in a manner similar to an indirect inguinal hernia but instead of passing downward a sac passes anteriorly between any two layers of the abdominal wall [2]. A Spigelian hernia is defined as a hernia occurring through the Spigelian aponeurosis. The herniation occurs through slit-like defects in the aponeurotic layer between the rectus abdominis muscle medially and the semilunar line laterally (so-called Spigelian fascia) [3]; it constitutes less than 2% of all hernias and is reported that more than 90% lie in the “Spigelian belt”[4]. Intraparietal inguinal hernias mimic Spigelian hernias clinically; although its treatment is very simple but pre-operative diagnosis is really a challenging issue [2]. We treated an old patient woman with a rare variant of the inguinal hernia, a giant interstitial intraparietal hernia misdiagnosed as a Spigelian hernia.

## Patient and observation

**Figure 1:**
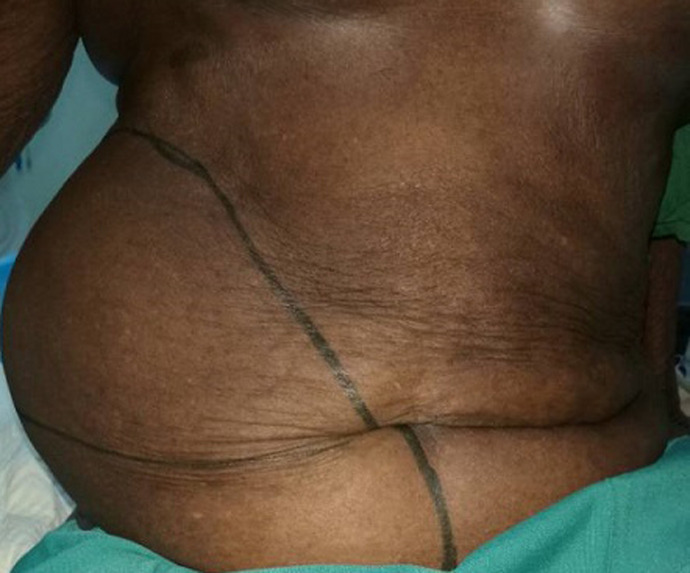
giant intraparietal inguinal hernia (front view)

**Figure 2:**
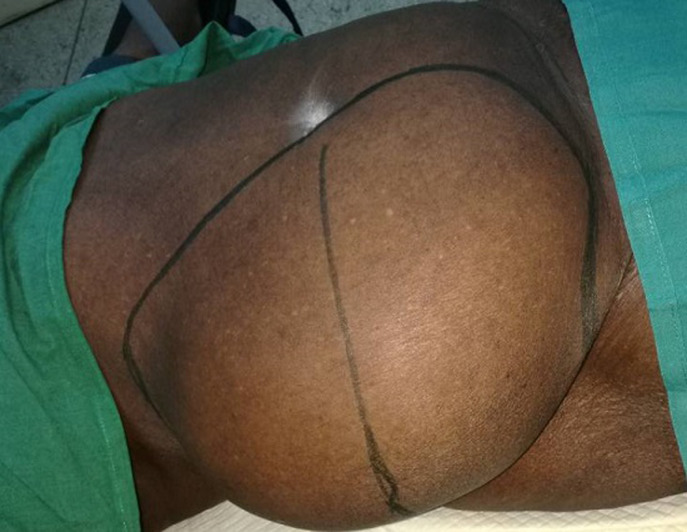
giant intraparietal inguinal hernia (lateral view)

An 85 years old black woman presented with complain of a large swelling over right abdomen for around 25 years. The swelling increased progressively over the years and it is associated with post-prandial discomfort. Her bowel habits were normal and there was no urinary complain. There was no history of trauma or any surgical procedure in the past. Her past medical history included hypertension for the last 10 years ago with regular treatment. Her physical examination revealed a huge mass of 25 x 30 cm occupying right flank and right lumbar region extending up to the umbilicus and inguinal region. Cough impulse was present. The mass was round shaped, soft, mild tender and partially reducible with gurgling sound (Figure 1, Figure 2). Percussion notes were resonant, and bowel sounds were present over the mass. A clinical impression of a giant Spigelian hernia was made. Laboratory investigations were made, all within the normal limits. An abdominal ultrasonography was performed. The ultrasonogram report suggested the probability of Spigelian hernia and advised CT scan for confirmation. CT scan was not done because of financial limitation of the patient. The final diagnosis of Spigelian hernia was made. After establishing the diagnosis of Spigelian hernia operative open intervention was planned.

**Figure 3:**
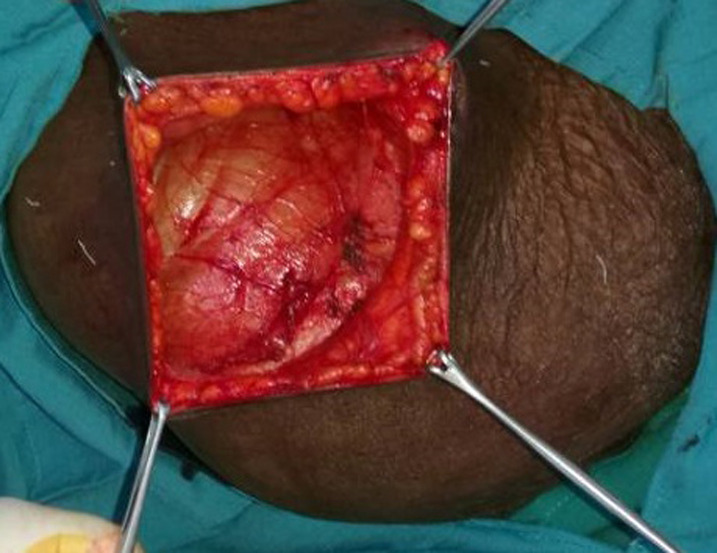
hernia sac laying below the external oblique fascia

**Figure 4:**
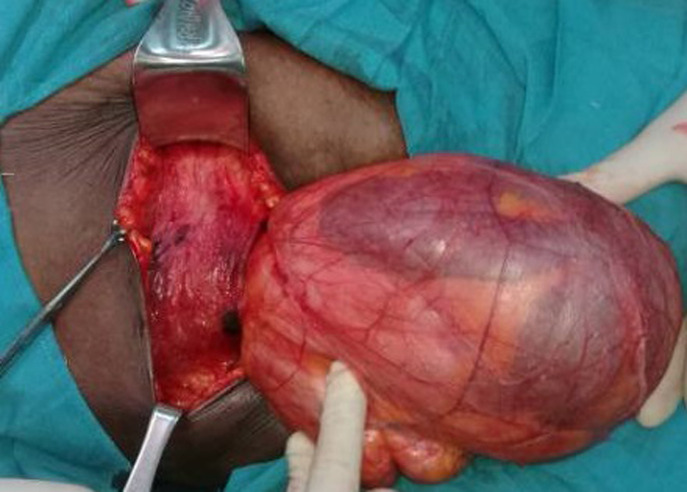
dilated deep inguinal ring

**Figure 5:**
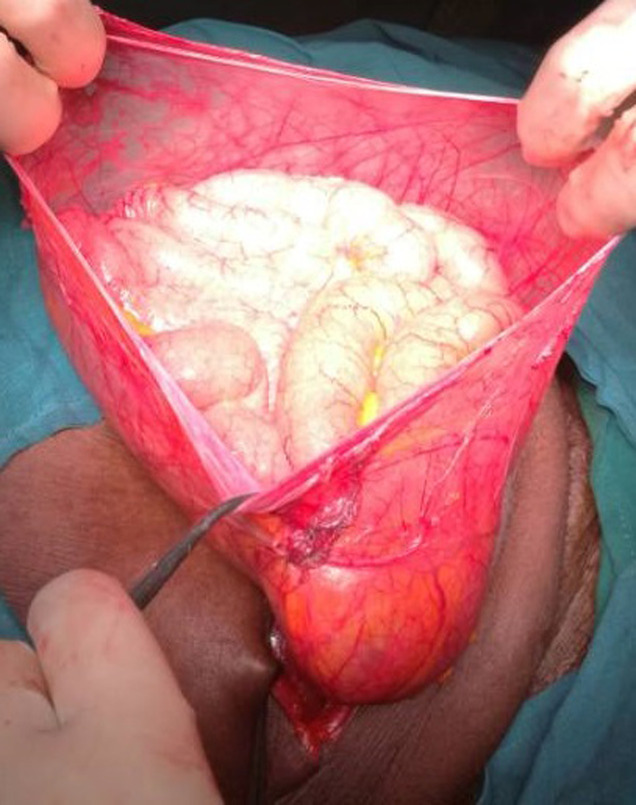
small bowels as content of the sac

**Figure 6:**
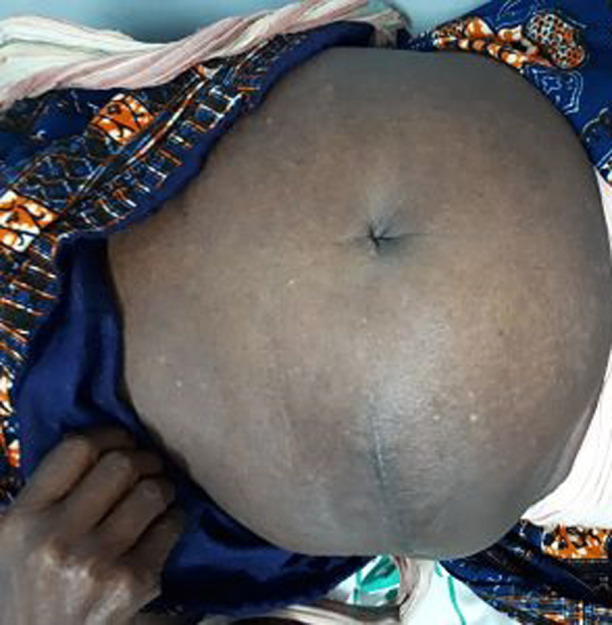
abdomen after 6-months post operation

Intraoperatively it was found to be intraparietal inguinal hernia. Surgery started with transversal incision over the mass, after external oblique fascia opened careful exploration revealed that the hernia sac was lying in between external & internal oblique muscles; the hernia sac was coming out of a very widely dilated deep inguinal ring of about 5 cm diameter (Figure 3, Figure 4) and extending forward superiorly and laterally as well as medially. A long viable segment of the small bowel with their mesentery was the content of the sac (Figure 5). The hernia sac was ligated at the deep inguinal ring as in traditional inguinal herniorrhaphy. A polypropylene mesh placement at the posterior wall of the inguinal canal was done. Post-operative period was uneventful and the patient was discharged on third postoperative day, she has now been on our follow-up for the last 6 months and is doing well (Figure 6). Informed con-sent was taken from the patient for the publication of this case report and accompanying images.

## Discussion

Intraparietal inguinal hernias form a group of rather unusual hernias located between the layers of the abdominal wall in the inguinal region [2,3]. The incidence of intraparietal hernias reported in three different studies is about 0.01-1.6% [5,6] and 0.08-1.6% [7]. It Have been described three different subtypes; they are preperitoneal (between peritoneum and transversalis fascia), interstitial (between transversalis fascia and transverse, internal oblique or external oblique muscles), and superficial (between external oblique and skin or within aponeurosis of the inguinal region) [5,6]. We presented a case of an interstitial variety, which is the most common of the three subtypes, comprising around 60 % [5,7]. In adults, the interstitial inguinal hernia is generally found after the four decades of life and the clinical diagnosis will be easy normally in the present of a large inguinal-iliac swelling [6]; however, accurate diagnosis of this anatomical type is rarely made preoperatively [6,8]. Another difficulty is the likelihood of confusing this anatomical variety of inguinal hernia with a Spigelian hernia [6] as in our case. Unspecific and variable presentation makes it liable for misdiagnosis preoperatively [9]. Lower Spigelian hernias are rare and should be differentiated from direct inguinal hernias and supravesical hernias [10]. CT and ultrasound have been used as a radiological adjunct for diagnosis in doubtful cases [11-13]; however, CT scan is issued as a gold standard diagnostic modality in the doubtful cases [14]. The low socio-economic situation of our patient was a limitation in our case, being impossible to carry out this study.

The clinical presentation with a partially reducible mass and the location of the mass in our patient mimicked a Spigelian hernia. Although diagnosis may be challenging but treatment is simple surgical exploration. The approach of surgical exploration may be laparoscopic or open depends upon the surgeon´s choice and the facility available in hospital [14]. In our case the option was open surgery. The final diagnosis of the intraparietal inguinal hernia was made only after surgical exploration of the swelling with excision of the large hernia sac and mesh hernioplasty. Though intraparietal hernias have been reported by many authors but a case with the giant intraparietal hernia in an old woman is a very rare event with very little reported in medical literature.

## Conclusion

We treated a patient with an interstitial intraparietal inguinal hernia that mimics a Spigelian hernia because of the huge size, anatomy location and ultrasonographic findings. The impossibility of performing a CT scan of the abdomen made more difficult to reach an accurate preoperative diagnosis. While clinical diagnosis may be challenging the treatment is always the surgical exploration; once the surgical anatomy is comprehended, the definitive diagnosis is determined and the surgical repair is simple as in our case.
